# Structural elucidation of *Tsukamurella pulmonis* neutral polysaccharide and its visualization in infected mouse tissues by specific monoclonal antibodies

**DOI:** 10.1038/s41598-018-29864-y

**Published:** 2018-08-01

**Authors:** Adnan Saeed, Mariola Paściak, Sabina Górska, Ireneusz Ceremuga, Elżbieta Gamian, Piotr Ziółkowski, Marek Drab, Andrzej Gamian

**Affiliations:** 1 Department of Immunology of Infectious Diseases, Hirszfeld Institute of Immunology and Experimental Therapy, Polish Academy of Sciences, Weigla 12, 53-114 Wroclaw, Poland; 2 Department of Medical Biochemistry, Wroclaw Medical University, Chałubińskiego 10, 50-368 Wroclaw, Poland; 3 Department of Pathology, Wrocław Medical University, Marcinkowskiego 1, 50-368 Wrocław, Poland; 4 USI, Unit of Nanostructural Bio-Interactions, Hirszfeld Institute of Immunology and Experimental Therapy, Polish Academy of Sciences, Weigla 12, 53-114 Wroclaw, Poland; 5 Wrocław Research Center EIT+, Stablowicka 147, 54-066 Wrocław, Poland

## Abstract

*Tsukamurella pulmonis* is an opportunistic actinomycetal pathogen associated with a variety of rarely diagnosed human infections. In clinical cases of infection, *T. pulmonis* usually accompanies other bacterial pathogens. Because of these mixed infections, a robust diagnostic assay is important. The bacteria cell surface polysaccharides are considered not only useful targets for diagnostics but also intriguing subjects for analysis of the interactions that regulate the host response in general. Here, the structure of the polysaccharide component of the *T. pulmonis* cell wall was established. Sugar and methylation analysis and 2D-NMR techniques revealed that its polysaccharide belongs to the class of arabinomannan composed of branched tetrasaccharide repeating units, with addition of linear →6)-α-D-Man*p*-(1→ mannan. Rabbit polyclonal sera against *T. pulmonis* and *T. paurometabola* bacterial cells revealed cross reactivity between their antigens. Tissue samples from mice infected with *T. pulmonis* revealed liver abscesses and pathologic granules located intracellularly when immunohistochemically stained with monoclonal antibodies raised against *T. pulmonis* polysaccharide. Ultrastructural studies revealed that these granules contain *T. pulmonis* cells. These observations indicate that *T. pulmonis* is a pathogenic species capable of spreading within the organism, presumably through the blood.

## Introduction

*Tsukamurella* strains are opportunistic bacteria and are often accompanied by other pathogens. These strains are increasing worldwide, but it is still difficult to estimate their morbidity. The bacteria cause severe infections in immunocompromised humans with chronic pulmonary disease, HIV infection or long-term indwelling central venous catheters^1,2^. Bacteria of the genus *Tsukamurella* are aerobic, Gram-positive rods. They are partially acid-fast non-motile bacilli that belong to the order *Actinomycetales* in the class *Actinobacteria*. The first description of *Tsukamurella* was reported in 1971 by the Japanese authors Tsukamura and Mizuno, who isolated a new organism, *Gordona aurantiaca*, from the sputum of a patient suffering from chronic pulmonary disease^3,4^. *Tsukamurella* was established as a separate genus by Collins *et al*.^5^. Species in the *Tsukamurella* genus share many phenotypic features with other species in genera such as *Corynebacterium*, *Nocardia*, *Rhodococcus* and *Mycobacterium* and might be misidentified as one of these species^6^. Thus far, the genus *Tsukamurella* includes 14 species isolated from medical cases and the environment, including *Tsukamurella paurometabola*^5^, *T. pulmonis*^7^, *T. tyrosinosolvens*^8^, *T. pseudospumae*^9^, *T. spongiae*^10^, *T. inchonensis*^11^, *T. strandjordii*^6^, *T. soli*^12^, *T. spumae*^13^, *T. sunchonensis*^14^, *T. carboxydivorans*^15^, *T. hongkongensis* and *T. sinensis*^16^, and *T. serpentis*^17^. Recently, reclassification of species within the genus *Tsukamurella* has been proposed—*T. spongiae* should be reclassified as a later heterotypic synonym of *T. pulmonis*, *T. carboxydivorans* as a synonym of *T. tyrosinosolvens*, and *T. sunchonensis* as a later heterotypic synonym of *T. pseudospumae*. All these studies were based on phenotypic characteristics and on comparison of whole bacterial genomes^16^.

*T. pulmonis* was isolated from the sputum of a 92-year-old woman suffering from pulmonary tuberculosis^7^. *T. pulmonis* is a rare human pathogen associated with immunosuppressed patients, including oncologic ones^1,18,19^. A variety of clinical forms have been associated with these bacteria, such as pneumonia^20^, conjunctivitis^21^, keratitis^22^ and catheter-related bacteraemia. Since *T. pulmonis* in clinical isolates is usually found together with other pathogens, it was proposed that none of these species is capable of breaking the defence barriers alone; co-infecting pathogen seemed to be necessary to complete the invasion process.

Polysaccharides of bacterial cell surfaces have a broad range of functions in the ecology of microorganisms at interfaces of the bio-habitat environment and, for pathogens, in the tissue microenvironment of the host^23^. Polysaccharides are also considered useful targets for diagnostic strategies and vaccine development. Currently, the structure of the polysaccharide component of the *T. pulmonis* cell wall has not been published. The objective of this study was to determine the structure of the polysaccharide antigen from the cell wall of *T. pulmonis* and generate antibodies against this polysaccharide, which will be useful for studying bacterial infection of host tissue using immunohistochemical and electron microscopic identification of the distribution of polysaccharide epitopes both on the bacteria and within tissues infected with this pathogen.

## Results

### Strain identification by MALDI-TOF mass spectrometry

The reference strain of *T. pulmonis*, PCM 2578 (PCM-Polish Collection of Microorganisms), was assessed by the matrix-assisted laser desorption-ionization time-of-flight (MALDI-TOF-MS) Biotyper system. The reference database (Biotyper 3.1, 4613 entries) possessed only two representatives of the *Tsukamurella* genus—*T. paurometabola* and *T. inchonensis*. Initially, the MALDI-TOF-MS Biotyper identified the *T. pulmonis* PCM 2578 reference strain as *T. paurometabola* or *Tsukamurella* sp. However, the scores obtained independently of culture conditions, ranging from 1.699–1.919, allowed for the recognition at the genus-level only. After database upgrading, the identification was correct at the species level (Table 1). We observed slight differences in score values obtained using different culture conditions.

**Table 1:** Identification of the reference strain *T. pulmonis* PCM 2578 in Biotyper and in in-house databases, respectively.

Strain/culture conditions^a^	Biotyper (Bruker)		In-house Database	
	Score value	Best match	Score value	Best match
PCM 2578/48 h-solid 79	1.763	*T. paurometabola*	2.817	*T. pulmonis*
PCM 2578/48 h-liquid 79	1.720	*Tsukamurella* sp.	2.118	*T. pulmonis*
PCM 2578/48 h-blood agar	1.919	*Tsukamurella* sp.	2.577	*T. pulmonis*
PCM 2578/48 h-nutrient agar	1.699	Not reliable	2.203	*T. pulmonis*

^a^Standard extraction procedure was performed according to the manufacturer^39^.

### Cell surface characterization

The cell surface properties related to the presence of polysaccharide and biofilm formation contribute to host surface adhesion and development of persistent infection. The yield of *T. pulmonis* PCM 2578 cell mass grown on 79 medium under aerobic conditions was 1.60 g/L. Analysis of the bacterial cell surface hydrophobicity of *T. pulmonis* PCM 2578 showed distinct hydrophobic surface properties, as presented in Table 2. Adhesion of bacterial cells to the glass test tube was estimated visually by observing the biofilm that covers the walls of test tubes after the incubation period. Adherence to the glass is correlated to hydrophobicity of cells of the *Tsukamurella* strains studied. The ability of the bacteria to adhere to a polystyrene surface did not significantly differ among the strains (Table 2).

**Table 2:** Cell surface hydrophobicity of the representatives of genus *Tsukamurella*.

Species	Measurement of hydrophobicity: [(λ1- λ2)]/ λ1*100%	Adherence to glass	Adherence to plastic [A_490_]
*Tsukamurella pulmonis* PCM 2578	59.76%	+	0.078
*Tsukamurella tyrosinosolvens* PCM 2579	75.60%	++	0.128
*Tsukamurella inchonensis* PCM 2577	74.13%	++	0.076
*Tsukamurella paurometabola* PCM 2453	91.15%	++	0.095

### Structure determination of polysaccharides

Polysaccharide (PS) was extracted from *T. pulmonis* bacterial mass by trichloroacetic acid, then precipitated by ethanol, and purified by enzymatic treatment and fractionation by ion-exchange chromatography on DEAE-Sephadex A-25 (DEAE-diethylaminoethyl). Polysaccharide not retained on the ion-exchange column was purified by gel filtration on a Toyopearl HW-55S system. The average yield of PS preparation from dry bacterial mass was 0.005%. Analysis of neutral sugars of the purified PS by GLC/MS revealed that it consisted of arabinose and mannose at a molar ratio of 1:3.5; trace amounts of Glc and Gal were also detected. The limitation of the method used was that it did not allow to detect acidic monosaccharides, therefore the presence of such compounds was excluded based on NMR analysis. Methylation analysis revealed the presence in *T. pulmonis* PS of 5-substituted arabinofuranose (2,3-di-*O*-methyl-1,4,5-tri-*O*-acetyl-arabinitol-1-d derivative) (2,3-Me_2_Ara), terminal mannopyranose (2,3,4,6-tetra-*O*-methyl-1,5-di-*O*-acetyl-mannitol-1-d), 2,5-disubstituted arabinofuranose (3-*O*-methyl-1,2,4,5-tetra-*O*-acetyl-arabinitol-1-d) (3-MeAra), 2-substituted mannopyranose (3,4,6-tri-*O*-methyl-1,2,5-tri-*O*-acetyl-mannitol-1-d), 6-substituted mannopyranose (2,3,4-tri-*O*-methyl-1,5,6-tri-*O*-acetyl-mannitol-1-d) and 2,6-disubstituted mannopyranose (3,4-di-*O*-methyl-1,2,5,6-tetra-*O*-acetyl-mannitol-1-d) in molar ratio approximately 1:2:3:6:2:1.

**Figure 1:**
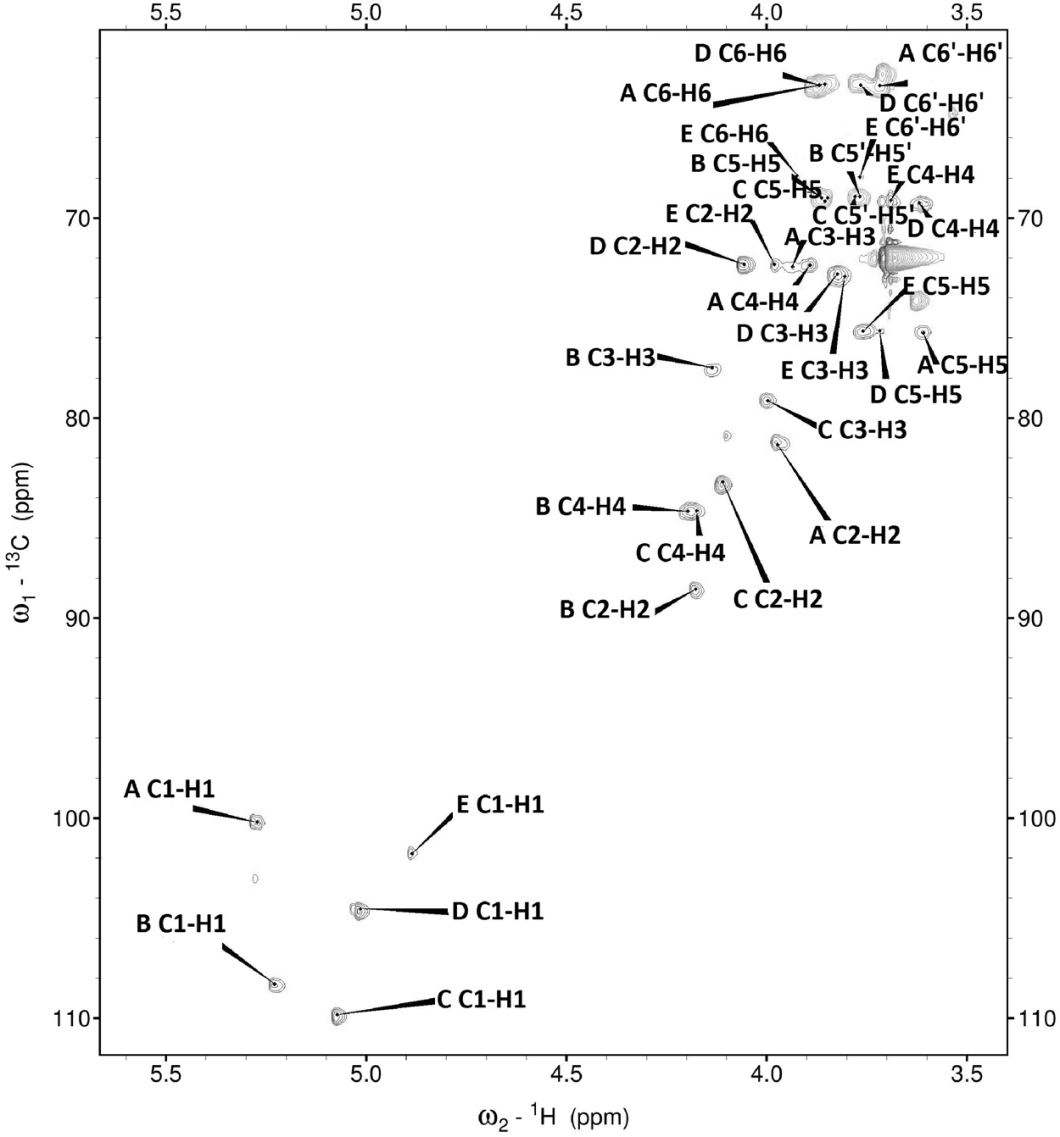
Selected portion of the ^1^H-^13^C-HSQC spectrum of the *T. pulmonis* polysaccharide. The corresponding parts of the ^1^H and ^13^C NMR spectra are shown along the horizontal and vertical axes, respectively. The letters refer to carbohydrate residues, and the Arabic numerals refer to proton/carbon in the respective residue denoted as shown in Table 3.

The ^1^H NMR and HSQC spectra recorded from the sample contained signals for five anomeric protons and carbons while signals of N-, O-acetyl, methyl and carboxyl groups were not detected. The major signals and spin-systems of five sugar residues were assigned by COSY (H-2), TOCSY (H-3, H-4, H-5), and NOESY (H-6) experiments. From the assigned ^1^H signals and the one-bond C-H connectivities, the carbon signals were assigned in the HSQC spectrum in which CH_2_ moieties of all the sugars were readily identified as negative cross-peaks. The monosaccharide residues in the sample were arbitrarily labelled A to E, according to decreasing chemical shift values of their anomeric protons (Fig. 1). Accounting for the published NMR data^24,25^ and comparing the chemical shifts for the individual monosaccharides, we identified the sugars and determined their anomeric configurations (Table 3). The substitution positions of the respective monosaccharides were identified based on the relatively high chemical shift values of the signals of the substituted carbons compared to values for the unsubstituted monosaccharides^26–29^.

**Table 3:** ^1^H and ^13^C NMR chemical shifts and selected inter-residue (HMBC) connectivities from the anomeric protons of *T. pulmonis* polysaccharide.

	Sugar residue	H1	H2	H3	H4	H5	H5′	H6	H6′	HMBC
		C1	C2	C3	C4	C5		C6		
A	→2)-α-D-Man*p*-(1→	5.27	3.97	3.93	3.89	3.61		3.72	3.85	**A1-B2 (88.5)** A1-A3, A1-A5
		100.2	81.3	72.4	72.3	75.7		63.3		
B	→2,5)-α-L-Ara*f*-(1→	5.23	4.17	4.13	4.19	3.86	3.76			**B1-C5 (68.9)** B1-B3, B1-B4
		108.3	88.5	77.5	84	68.9				
C	→5)-α-L-Ara*f*-(1→	5.07	4.11	3.99	4.17	3.85	3.77			**C1-B5 (68.9)** C1-C3, C1-C4
		109.8	83.2	79.1	84.6	68.9				
D	t-α-D-Man*p*-(1→/	5.01	4.06	3.82	3.62	3.72		3.86	3.76	**D1-A2 (81.3)** D1-D3, D1-D5
		104.5	72.3	72.8	69.2	75.6		63.3		
E	→6)-α-D-Man*p*-(1→	4.88	3.98	3.8	3.69	3.76		3.92	3.76	E1-E3 (weak signal)
		101.8	72.3	72.9	69.1	75.6		67.9		

Spectra were obtained for D_2_O solution at 25 °C. Acetone (δ_H_ 2.225, δ_C_ 31.05 ppm) was used as an internal reference.

Residue **A** with H1/C1 signals at δ 5.27/100.2 was identified as 2-substituted α-D-Man*p* based on the low chemical shifts of the C-2 signal (δ 81.3). Residue **B** with H1/C1 signals at δ 5.23/108.3 was recognized as 2,5-substituted α-L-Ara*f* due the characteristic low chemical shifts of the C-2 signal (δ 88.5) and C-5 signal (δ 68.9). Residue **C** with H1/C1 signals at δ 5.07/109.8 was recognized as 5-substituted α-L-Ara*f* due the characteristic low chemical shifts of the C-5 signal (δ 68.9). Residue **D** with the H-1/C-1 signals at δ 5.01/104.5 was recognized as the terminal α-D-Man*p* based on the proton and carbon chemical shifts typical of those of mannose; however, its chemical shifts were very close to the HOD peak and were sometimes lost on saturation of the HOD. Chemical shifts of this residue are similar to those of terminal mannose in *Mycobacterium bovis* arabinomannan, a polysaccharide with a similar molecular mass that was also analysed in D_2_O^30^. Residue **E** with the H-1/C-1 signals at δ 4.88/101.8 could be identified as the 6-substituted α-D-Man*p* residue from the low chemical shifts of the C-6 (δ 67.9); however, the chemical shifts were weak and hence not always visible in the spectra.

The pyranose ring of systems A and D was confirmed by the cross-peaks observed on the ^1^H-^13^C HMBC experiment between their H-1 and their respective C-3 and C-5. A weak cross-peak was also observed between H-1 and C-3 of residue E, indicating a pyranosidic form, whereas the furanose ring of systems B and C was confirmed by the cross-peaks between their H-1 and their respective C-3 and C-4.

**Figure 2:**
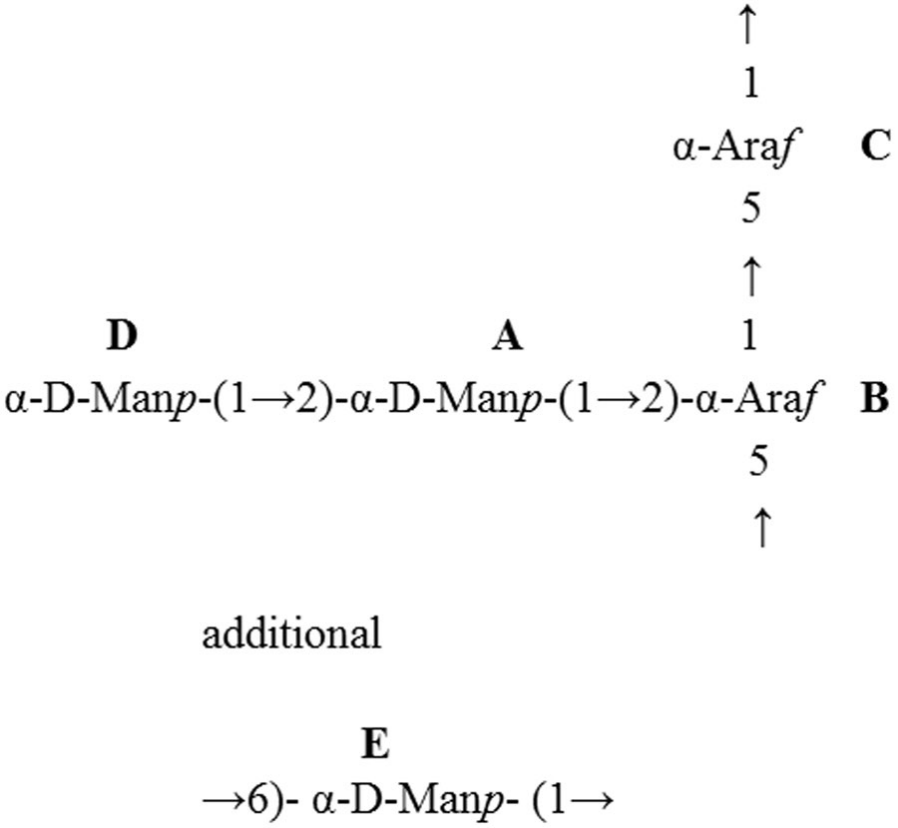
The structure of the tetrasaccharide repeating unit of the polysaccharide and linear mannan from *T. pulmonis* evaluated in this study.

The sequence of the monosaccharide residues within the repeating unit of the sample was determined through the assignment of the long-range couplings in the HMBC spectrum. The HMBC spectra showed cross-peaks between the anomeric proton and the carbon at the linkage position (Table 3). No correlation was observed between H-1 of residues A, B, C or D with C-6 of residue E, indicating that residues A, B, C or D do not substitute the E residue. Thus, the combined results show the tetra-saccharide repeating unit of the polysaccharide of *T. pulmonis* as in Fig. 2.

### Immunochemical analysis

**Figure 3:**
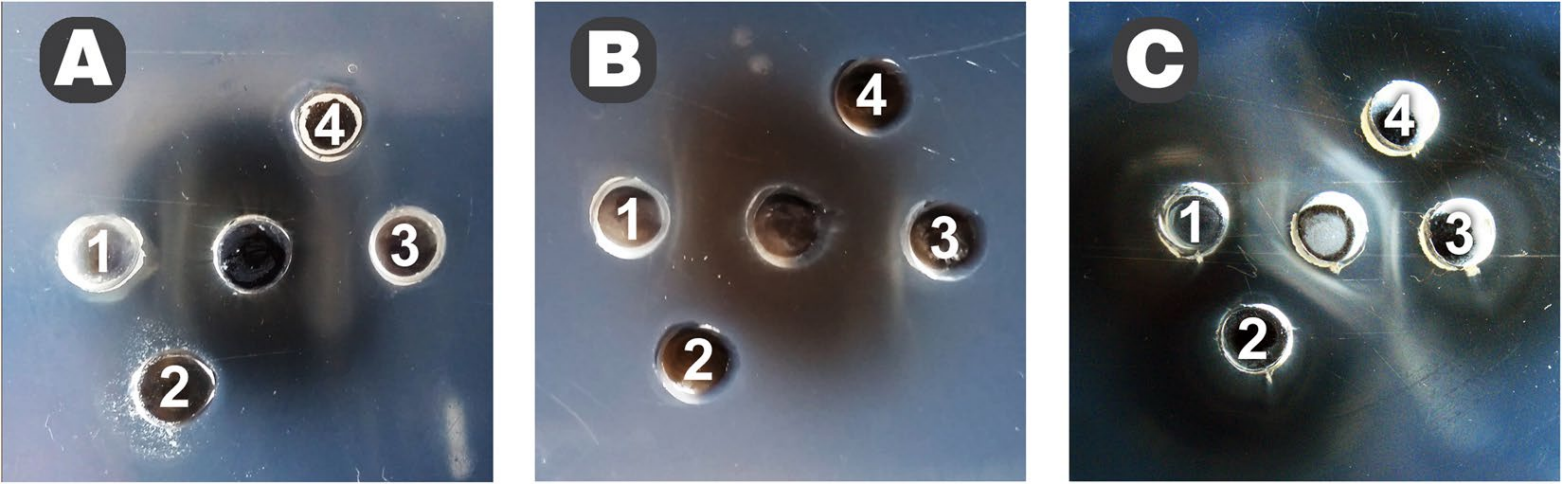
Double immunodiffusion of rabbit sera against whole cells of *T. pulmonis* (**A**) and *T. paurometabola* (**B**) with cells of *T. paurometabola* (1), *T. tyrosinosolvens* (2), *T. pulmonis* (3), *T. inchonensis* (4). Reactivity of *T. pulmonis* polysaccharide (**C**) with sera against whole cells of *T. pulmonis* (1), *T. tyrosinosolvens* (2), *T. inchonensis* (3), and *T. paurometabola* (4). The polyclonal rabbit sera against whole cells of *T. pulmonis* and *T. paurometabola* reacted specifically with homologous bacteria but not with cells of *T. tyrosinosolvens* and *T. inchonensis*. The cross-reactivity of polysaccharide antigen from *T. pulmonis* was observed with all studied *Tsukamurella* antisera.

**Figure 4:**
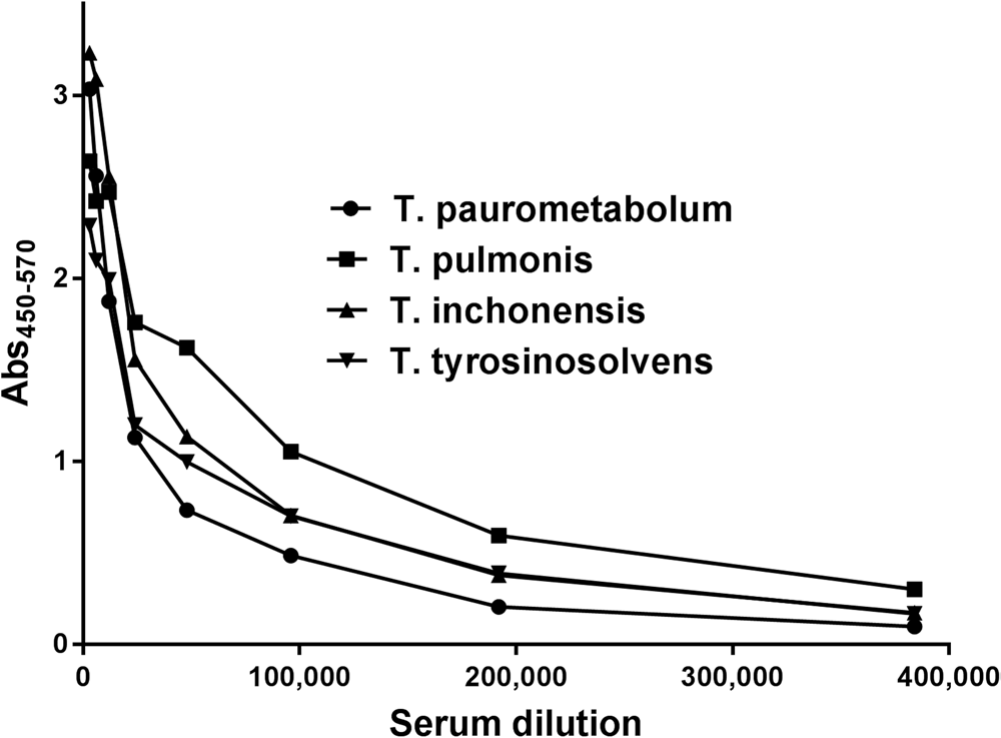
Reactivities in the ELISA test of polyclonal rabbit serum against *T. pulmonis* with *Tsukamurella* cells: *T. inchonensis*, *T. paurometabola*, *T. pulmonis*, and *T. tyrosinosolvens*. The cross-reactivity with all studied strains was observed; however, the highest antibody levels were with homologous bacteria.

**Figure 5:**
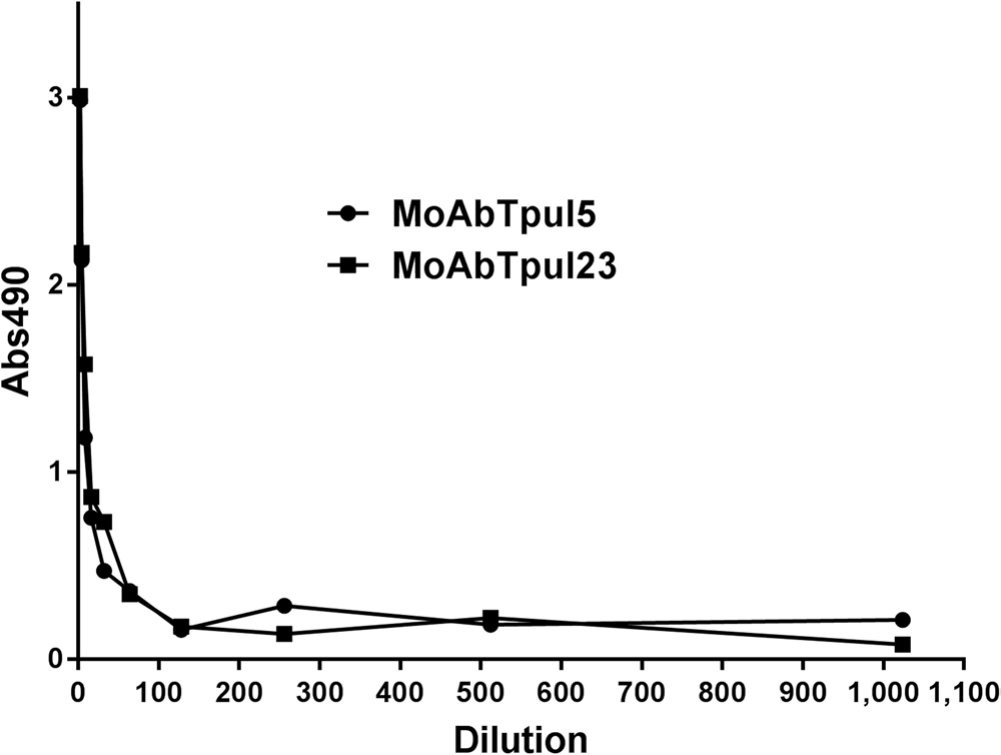
Reactivities in the ELISA test of monoclonal antibodies 5 and 23 (MoAbTpul5 and 23) against *T. pulmonis* polysaccharide with homologous antigen (PS of *T. pulmonis*). Both monoclonal antibodies reacted with polysaccharide from *T. pulmonis*.

We obtained rabbit sera against *T. pulmonis* and *T. paurometabola* cells. Interestingly, rabbit polyclonal serum against *T. pulmonis* cells reacted specifically with homologous bacteria and *T. paurometabola*, whereas *T. tyrosinosolvens* and *T. inchonensis* cells were not recognized by this serum (Fig. 3A); the same pattern of reactivity was observed with polyclonal rabbit serum against *T. paurometabola* cells (Fig. 3B). The results of immunodiffusion indicate the similarity of cell surface antigens of *T. pulmonis* and *T. paurometabola*, in contrast to *T. tyrosinosolvens* and *T. inchonensis*, and suggest that different epitopes are recognized by rabbit polyclonal antibodies. In contrast, enzyme-linked immunosorbent assay (ELISA) reactivity with four species of *Tsukamurella* was observed (Fig. 4). Furthermore, reactivity of the polysaccharide antigen of *T. pulmonis* with polyclonal sera against *T. pulmonis, T. tyrosinosolvens, T. inchonensis* and *T. paurometabola* cells in double immunodiffusion tests was observed (Fig. 3C), and cross reactivity of PS was observed with all *Tsukamurella* antisera studied. The next step was preparation of monoclonal antibodies specifically against *T. pulmonis* polysaccharide by the hybridoma technique. Two monoclonal antibodies were obtained, namely, MoAbTpul5 and MoAbTpul23, both of the IgM class (Fig. 5).

### Immunohistochemistry of mouse tissue infected with *T. pulmonis*

**Figure 6:**
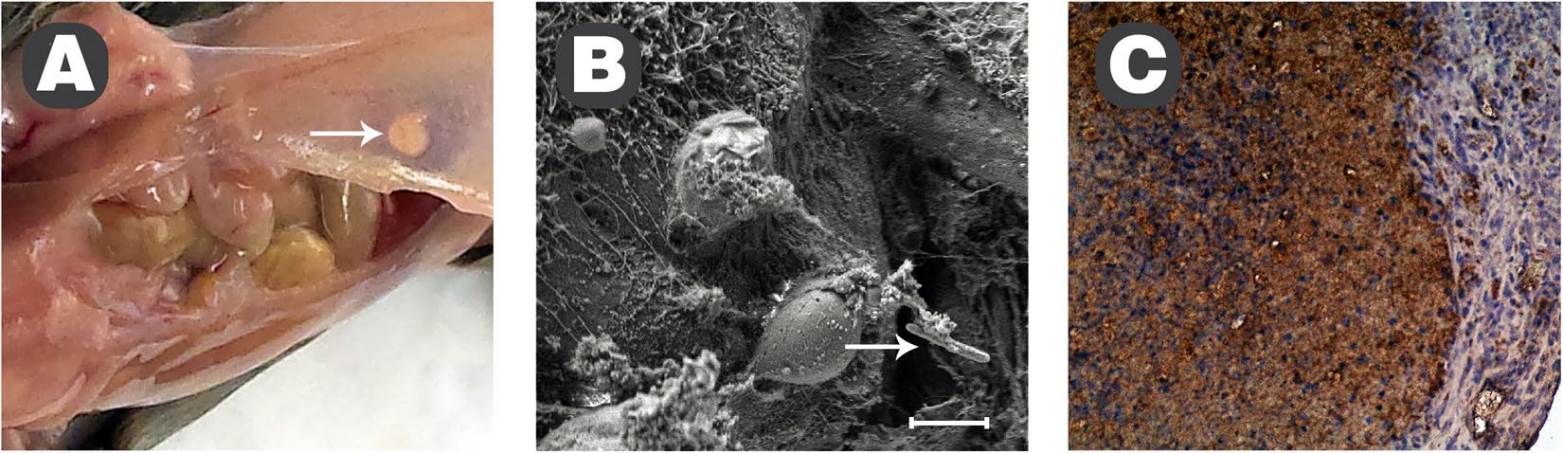
Visualization of *T. pulmonis* infection in mouse tissues. Abscess induction in the abdominal peritoneum three days after the cell injection (**A**) and electron micrograph showing a single bacterium spread to the liver tissue from the abdominal peritoneum (**B**). Reactivity of monoclonal antibody MoAbTpul23 in tissue, magnification 200x, present as brown, dot-like granules in inflammatory cells; LSAB, haematoxylin counterstained (**C**) Scale bar in (**B**) 2 µm.

To establish the mode of interaction of *T. pulmonis* cells with host tissues, specimens containing abscesses were evaluated with histological and immunohistochemical procedures. Macroscopically, we observed overall signs of disease-related distress in the animals. Gross anatomy during the autopsy of sacrificed animals demonstrated haemorrhagic pneumonia in the lungs. Three days after the injection of *T. pulmonis* bacterial cell suspension at a concentration of 1.5 × 10^12^ CFU ml^−1^, bacteria induced distinct abscesses in the mice, as shown in Fig. 6A,B. Tissue samples from infected mice with liver abscesses appeared to contain granules located intracellularly when stained with the monoclonal antibody MoAbTpul23 (Fig. 6C). We observed the same histological changes in the skeletal muscles (not shown). Positive reactions were observed in inflammatory cells within the entire abscess stained with MoAbTpul23 as brown, dot-like staining (centre and left-hand side, Fig. 6C).

**Figure 7:**
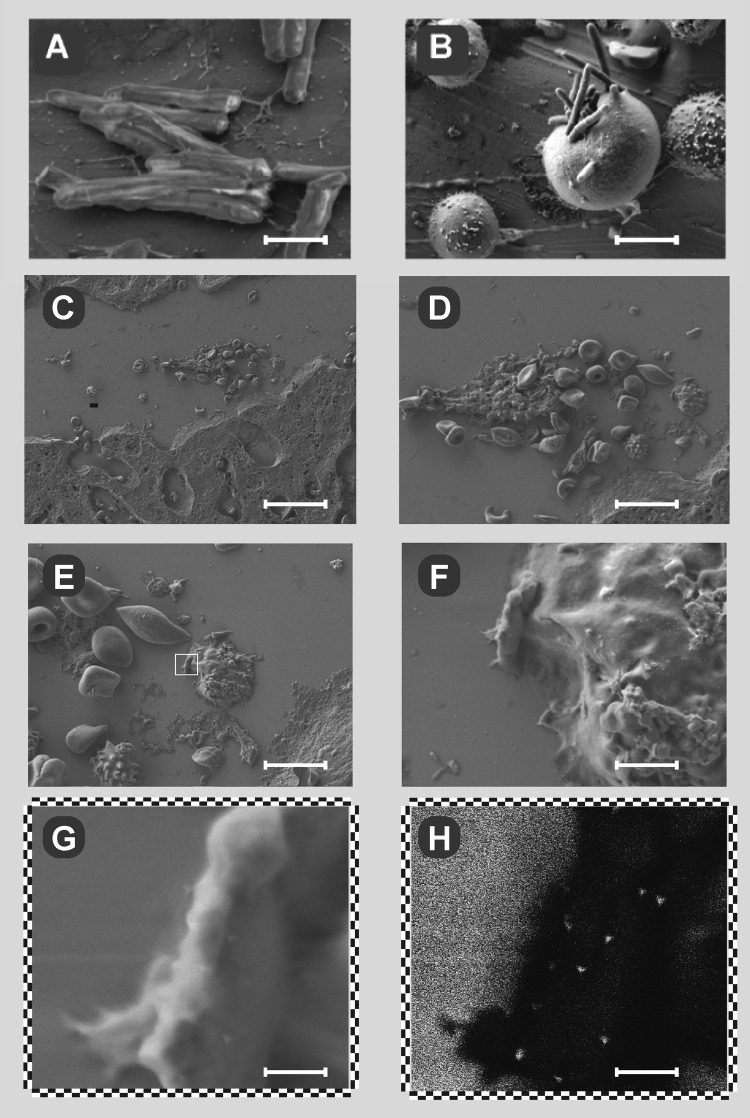
Electron micrographs of *T. pulmonis* PCM 2578 *in vitro* and *ex vivo*. *T. pulmonis* (**A**) an *in vitro* culture, and the uptake process during model infection of this pathogen by macrophage/splenocytes *in vitro* (**B**), and *ex vivo* (**C**-**H**), the latter shown in the mouse liver, as a series of increasing resolution. The C and D show lumen of small blood vessel with accumulation of leukocytes and blood platelets, besides erythrocytes and the surrounding tissue. As shown in inserts under larger magnifications (E to H) these are the foci surrounding the pathogen’s remnants. The insert in (**E**) is magnified in (**F**) and in (**G**-**H**) to demonstrate the immunostaining pattern of pathogens detected with the antibody against *Tsukamurella* PS; secondary antibody was labelled with 15 nm colloidal gold. (**H**) emphasizes the chemical contrast of immunogold (bright spots) on the bacterial body, captured during uptake by murine macrophages (dark-appearance), (EsB detector). Scale bar, 500 nm in (**A**), 2 µm in (**B**), 20 µm in (**C**), 10 µm in (**D**), 5 µm in (**E**), 1 µm in (**F**), 200 nm in (**G**) and (**H**).

These results indicate the actinomycosis type of localized abscess with numerous inflammatory cells. Immunohistochemical staining revealed granules inside the cells. To reveal the nature of these granulations, detailed investigations of tissues samples were performed with scanning electron microscopy (Fig. 7). The morphology of the *T. pulmonis* bacterial cells analysed by scanning electron microscopy showed uniform bacterial rods covered with a dense mucous structure of the polysaccharide coat, as presented in Fig. 7A. The electron micrograph of the cells shows the surface with its coat, made visible without metal deposition, directly, by low-voltage FESEM with an in-lens SE1 electron detector. During model infection *in vitro* (Fig. 7B), the uptake of this pathogen by macrophages/splenocytes could be observed. The macrophages were isolated from the spleen (splenocytes) of 6-week-old mice, as described previously^31^. The uptake of *T. pulmonis* bacteria by macrophages (splenocytes) was performed for 60 min. This uptake of the pathogenic bacteria by macrophages was vigorous and almost completed after 1 h (Fig. 7B). The bacteria could be observed in various stages of uptake by macrophages (in the centre of the view field), from the initial stage (back of the cell), to halfway through the process (left aspect of the cell) to complete phagocytosis (right of the cell and top). Thus, the polysaccharide coat does not protect the *T. pulmonis* bacteria from phagocytosis even in the absence of opsonins. An incident beam of low accelerating voltage allows low landing energies that enable direct detection mode, and no coating is necessary. *In vivo* infection of mice with *T. pulmonis* is shown in the liver as a series of increasing resolution in Fig. 7C–H. The insert in (E) is magnified in (F) and (G-H) to demonstrate the immunostaining pattern of the pathogen detected with the antibody raised against *T. pulmonis* polysaccharide (MoAbTpul23); the secondary antibody used was labelled with 15 nm colloidal gold. Figure 7G shows the topography with the imaging procedure by an SE2 ET detector, similar to B-F, while (H) emphasizes the chemical contrast of immuno-gold (bright spots) on the bacterial body, captured during uptake by murine macrophages (dark-appearance), with use of energy-selective detection of back-scattered electrons (EsB detector), as described previously^32^. Together with the previously presented immunohistochemical data, these data demonstrate that *T. pulmonis* is capable of breaching defence barriers as a single infecting agent and of spreading to remote organs even in the absence of other co-infecting pathogens.

## Discussion

Members of the *Tsukamurella* genus are known as opportunistic pathogens and are agents of lung cavitations and tuberculosis-like syndromes^33^. Human infections caused by *Tsukamurella* spp. are rarely diagnosed, with the most common one being indwelling device-related, including catheter-related, bacteraemia^7^. Less frequently, other infections related to the respiratory tract^34^, peritonitis^35^ and brain abscesses are observed^36^. *T. pulmonis* and related nocardioform actinobacteria may be underdiagnosed or misdiagnosed as a cause of significant infections due to difficulties in identification by phenotypic procedures^33,37^. Aerobic actinomycetes are morphologically similar, for example *Nocardia*, *Tsukamurella* or fast growing mycobacteria, and are difficult to distinguish. Sometimes even 16S rRNA doesn’t allow to distinguish among these bacteria, but analysis of a region of the secA1 gene was shown to be suitable for discrimination of clinical species of *Tsukamurella*^19^. Moreover, whole genome comparison allows reclassification of newly described species, which indirectly indicates overall similarity between representatives of this genus^38^. MALDI-TOF-MS has started replacing existing practices of microbial identification in clinical diagnosis. This technique, similar to other automated microbial identification systems, also relies on a reference databases for identification of microorganisms. As we have shown, an upgraded MALDI-TOF Biotyper database containing *Corynebacterineae* representatives was created and used for identification of the strain isolated from a nocardial brain abscess of an immunocompetent patient^39^. Here, we confirmed that upgrading of commercial database with few *Tsukamurella* species improves identification of *T. pulmonis*.

The cell surface properties related to the biofilm formation contribute to host surface adhesion and development of persistent infection. These factors should be elucidated for *T. pulmonis* to better understand the mechanisms of infection, pathogen carriage and immunity. Regarding cell surface hydrophobicity measured with two tests, four species of *Tsukamurella* have hydrophobic surfaces, and no difference was observed in adherence to polystyrene. Hydrophobicity of bacterial cell surface depends on biofilm and polysaccharides as well as surface proteins, which might contribute, in different ways, to the adherence to glass and plastic surfaces.

Pathogenicity of several bacteria depends on the presence and structure of their extracellular polysaccharides; therefore, *T. pulmonis* PS was subjected to structural elucidation. The polysaccharide is an arabinomannan composed of branched tetrasaccharide repeating units where the →)-αAra*f*(1 → 5)-αAra*f*(→main chain is substituted by αMan*p*(1 → 2)αMan*p*(1 → 2)- disaccharide, with additional low amounts of linear →6)-α-D-Man*p*-(1→ mannan. This structure has been described as well in *T. paurometabola*, with a molecular mass of 12.5 kDa and mannan linked to a phosphatidylinositol anchor forming lipoarabinomannan^40^. This TpaLAM was found to induce TNFα via TLR-2. We used different extraction and purification procedures and cannot exclude the isolation of part of the lipoarabinomannan of *T. pulmonis*. Both species belong to the same genus and are highly related.

Monoclonal antibodies obtained against PS of *T. pulmonis* were found to be specific to *T. pulmonis*, while polyclonal rabbit sera cross-reacted with *T. paurometabola* and not with *T. tyrosinosolvens* and *T. inchonensis* cell extracts. The polysaccharide TpuPS was recognized by rabbit sera against *T. pulmonis*, *T. paurometabola*, *T. tyrosinosolvens* and *T. inchonensis*. This indicates partial cross-reactivity of antigens present in these species.

Mice exposed to intraperitoneal infection of *T. pulmonis* demonstrated clinical signs of disease, which was further confirmed by haemorrhagic pneumonia and abscesses of abdominal organs, as observed during autopsy. Histochemical and ultrastructural studies revealed severe pathologic inflammation of internal organs, whose aetiology was confirmed by the immunohistochemical results of strong reactivity of infection foci to monoclonal antibodies against *T. pulmonis*. These clinical and histopathological observations indicate that *T. pulmonis* is a pathogenic species with fulminant infection when localized in internal cavity organs. In human clinics, this pathogen is usually accompanied by other bacterial species, and thus, our results suggest that the permissive role of co-infecting bacteria that penetrate tissue barriers for *T. pulmonis* may be not necessary; *T. pulmonis* appeared to be capable of penetrating defence barriers and spreading to remote organs on its own. The strong infectious properties of *T. pulmonis* upon injection that were demonstrated in this study do not exclude the relevance of the multistep mechanism of the disease in which other bacteria prepare the penetration channels for *Tsukamurella*. However, robust diagnostic assays should be further developed to unequivocally identify *T. pulmonis* among other co-infecting species, since it should be eradicated as a potential cause of severe infection. Thus, the clinical relevance of *T. pulmonis* may be often overlooked and masked by other bacteria, while the direct pathogenic effects are caused directly by *Tsukamurella*; therefore, the monoclonal antibodies developed in this study against *T. pulmonis* polysaccharide may be an important aid in early diagnosis and monitoring of treatment efficacy.

## Methods

### Bacterial strains and cell mass preparation

*T. pulmonis* strain PCM 2578 (JCM 10111 ^T^), *T. paurometabola* PCM 2453 (ATCC 8368 ^T^, IMET 11082), *T. inchonensis* PCM 2577 (JCM 10110 ^T^), and *T. tyrosinosolvens* PCM 2579 (JCM 10112 ^T^) were obtained from the Polish Collection of Microorganisms at the Hirszfeld Institute of Immunology and Experimental Therapy. Bacteria were cultivated in liquid 79 medium (bacto-peptone, yeast extract with 2% glucose) under aerobic conditions with shaking at 37 °C for 48 h. Cells were harvested by centrifugation at 7500 rpm (4 °C, 20 min) and washed twice with PBS and once with MiliQ water and then lyophilized.

### MALDI-TOF mass spectrometry

For MALDI-TOF analysis *T. pulmonis* colonies cultivated on solid and liquid 79 medium and for comparison on nutrient and blood agar at 37 °C for 2 days were used. For the protein extraction method, the standard procedure recommended by the manufacturer^39^ was used. Protein extracts were smeared on an MTP 384 target plate with polished steel, dried, and coated with α-cyano-4-hydroxycinnamic acid matrix solution^39^. MALDI-TOF analysis was performed on the Ultraflex mass spectrometer (Bruker Daltonics, Germany) using the Biotyper software (version 3.1) and a database containing 4613 entries. Spectra were recorded in the linear positive mode at the laser frequency of 200 Hz within a mass range of 2000–20 000 Da. For the Biotyper database upgrade, the spectra of four reference *Tsukamurella* strains (*T. pulmonis* PCM 2578, *T. paurometabola* PCM 2453, *T. inchonensis* PCM 2577 and *T. tyrosinosolvens* PCM 2579) were additionally incorporated. The selected 20 spectra were used to create a reference Main Spectrum Profile (MSP) using the Bruker Biotyper 3.1 software.

### Measurement of the cell surface hydrophobicity

Surface hydrophobicity was estimated by a salt aggregation test of bacterial cells, which characterized the ability of bacteria to interact with a non-polar organic solvent, xylene. *Tsukamurella* spp. were grown in liquid 79 medium, and the cell suspensions were mixed with 0.1 M ammonium sulphate, centrifuged (6000 rpm, 4 °C, 15 min), and then washed three times with PBS. The bacterial pellet was suspended in 1.2 ml of PUM buffer, and the absorbance was measured at a wavelength of 405 nm. Then, 0.2 ml of xylene was added and was shaken vigorously for 2 min after a 10 min incubation. After 15 min, when both phases were again separated, the absorbance of the aqueous phase was measured at 405 nm^41,42^).

### Analysis of adhesiveness to the glass and polystyrene

The bacterial ability to adhere to the glass surface was estimated visually by observing the biofilm, which covered the walls of test tubes after the incubation period. *Tsukamurella* spp. were grown in glass tubes in 79 medium at 37 °C for 2 days. Then, the medium was decanted, and the tubes were filled with 3 ml of crystal violet solution for 60 seconds. Excess crystal violet was decanted, and then, the tubes were inverted and dried at room temperature. The density of biofilm covering the walls was estimated by referring to a semiquantitative scale (−, +, ++, +++)^43^.

A similar procedure was applied for measuring the adherence to a polystyrene plate, and the absorbance was read at 490 nm with a microplate reader^43^.

### Extraction and purification of the polysaccharide

Isolation and purification of *T. pulmonis* polysaccharides were performed according to^44^ Górska-Frączek *et al*.^44^. Briefly, *T. pulmonis* was extracted with 10% trichloroacetic acid, centrifuged and precipitated with 5 volumes of ice-cold 96% ethanol.

### Sugar and methylation analysis

Samples of 0.5 mg of the purified polysaccharide were hydrolysed (10 M HCl, 80 °C, 25 min) and then evaporated under a stream of nitrogen, and reduction was performed by adding 100 µl of 1 M NH_4_OH and 0.5 ml of a solution of NaBH_4_ in DMSO (10 mg/ml). Samples were incubated at 4 °C overnight, and then, 100 µl of 80% acetic acid was added to each sample. Acetylation was performed by adding 100 µl methylimidazole and 0.5 ml of acetic anhydride to each sample^45^. After a 15 min incubation at room temperature, three cycles of sugar derivative extraction were carried out with water-dichloromethane (1:1, v/v). The organic phase was dried in a stream of nitrogen and analysed by GLC-MS. For methylation analysis, the polysaccharide sample (0.3 mg) was permethylated according to^46^. The product was hydrolysed with HCl (10 M, 80 °C, 25 min), reduced with NaBD_4_ and acetylated as described above. Partially methylated alditol acetates were analysed by GLC-MS, which was performed on a Thermo Scientific ITQ 700 Focus gas-liquid chromatograph-mass spectrometer equipped with a Zebron column ZB-5HT (30 × 0.25 mm × 0.25 μm w/5 m Guardian). A temperature programme of 150–270 °C at 12 °C/min was applied. The partially methylated alditol acetates were identified based on their retention times and cleavage profiles^47^.

### NMR spectroscopy

The NMR spectra were obtained on a Bruker 600 MHz Avance III spectrometer using a 5 mm QCI ^1^H/^13^C/^15^N/^31^P probe equipped with a z-gradient. The NMR spectra were obtained for D_2_O solution of the sample at 25 °C using acetone (δ_H_ 2.225, δ_C_ 31.05 ppm) as an internal reference. The sample (3 mg) was repeatedly exchanged with D_2_O (99.9%) with intermediate lyophilization. The data were acquired and processed using Bruker Topspin software (version 3.1) and the SPARKY programme^48^. The signals were assigned using one- and two-dimensional experiments, COSY, TOCSY, NOESY, HSQC, with and without carbon decoupling, and HMBC. The TOCSY experiments were carried out with mixing times of 30, 60 and 100 ms and the NOESY experiments with mixing times of 100 ms and 300 ms. The ^1^H,^13^C HMBC spectrum was recorded with a 60-ms delay for the evolution of long-range spin couplings.

### Ethical statement

All animal experiments were performed according to EU Directive 2010/63/EU for animal experimentations and were approved by the 1st Local Committee for Experiments with the Use of Laboratory Animals, Wroclaw, Poland (no. 53/2009, 23/2012, 102/2016). The animals were bred under specific pathogen-free (SPF) conditions in the Animal Breeding Centre of the Institute of Immunology and Experimental Therapy (IIET).

### Preparation of monoclonal antibody

Six-weeks-old female mice of BALB/c were immunized three times with *T. pulmonis* cell suspension in PBS (optical density 1) emulsified with Freund’s adjuvant at a ratio of 1:1. The first dose (100 μl) was administered subcutaneously in the inguinal lymph node regions. After 30 days, the mice were immunized intraperitoneally with 50 μl of cell suspension without adjuvant, twice, with two week intervals. Three days prior to spleen collection, the mice received a booster dose of the antigen. The fusion of the spleen cells with SP-2/0 myeloma cells (plasmocytoma cells) was implemented according to the Köhler and Milstein procedure^49^. Screening was performed by 96-well MaxiSorp (Nunc) plates coated with *T. pulmonis* polysaccharide at 4 °C overnight by 1 µg/100 µl solution. Plates were washed twice with TBS-T, and then, 200 µl of 1% BSA in PBS was added to block non-specific binding. After washing, anti IgG-IgM-IgA antibodies (1:2000 in TBS) were applied, followed by washing steps. *O*-phenylene-diamine substrate was applied at 22 °C for 10 min. To stop the reaction, 50 µl of 2 M H_2_SO_4_ was added, and the plates were analysed at 490 nm on a Biotek instrument^50^. Immunoassay of immunoglobulin isotype was performed using an ELISA kit for determination of antibody classes (RD Biotech).

### Preparation of rabbit polyclonal immune sera

Rabbits were immunized three times with *T. pulmonis, T. paurometabola*, *T. tyrosinosolvens* and *T. inchonensis* cell suspensions in PBS (2 mg/ml), emulsified with incomplete Freund’s adjuvant (IFA) at a ratio of 1:1. The first dose (1 mg) was administered subcutaneously in 10 sites. The second and third doses with the same amount of antigen were injected at 2 week intervals. Rabbits were bled on the 10^th^ day after the last vaccination. Sera were depleted of complement at 56 °C for 30 min, and aliquots were stored at −20 °C.

Double immunodiffusion tests were performed on glass slides according to^51^.

### Infection of mice with *T. pulmonis*

Prior to injection, *T. pulmonis* bacteria were aerobically grown in liquid 79 medium at 37 °C for 2 days and then centrifuged, washed with PBS and diluted to an optical density 0.2. The suspension density was determined by counting the CFU on 79 medium plates. Female DBA mice (aged 3 months) were injected intraperitoneally with 500 µl of cell suspension. Three days after infection necropsy was performed to determine the presence of the abscesses, which were collected aseptically and fixed in 4% paraformaldehyde^52^. The samples were then processed for immunohistochemical analysis and immuno-gold labelling with monoclonal antibody raised against *T. pulmonis* polysaccharide.

### Histopathology and immunohistochemistry

The mouse tissue samples of the peritoneum and liver were fixed in 4% buffered paraformaldehyde. Paraformaldehyde-fixed, paraffin-embedded (FFPE) tissue sections were cut from blocks into 4 µm slices, mounted on poly-L-lysine-coated glass slides, deparaffinized by heating at 90 °C and then immersed in xylene for 9 min. Sections were stained with haematoxylin and eosin (HE) or, alternatively, the immunoperoxidase technique was performed with the ABC DAKO kit. Briefly, the following steps were performed: endogenous peroxidase was blocked with the blocking reagent; then, sections were placed in distilled water at room temperature (15 min); citric acid buffer of pH 6.0 (2 × 8 min, heating in microwave Daewoo at 350 Watt and at room temperature) was applied; then, TBS (0.05 mM), pH 7.6 with swine serum at 1:50 (0.5 h at room temperature) was used; the samples were washed in distilled water; the monoclonal antibody against polysaccharide of *T. pulmonis* (150 µl/slide, 40 °C, overnight) was applied; samples were washed in TBS; LSAB reagent (30 min) was used; and 3,3′-diaminobenzidine tetrahydrochloride (DAB) was applied for 5 min. The slides were counterstained with haematoxylin and mounted under coverslips with resin. Negative controls were carried out with TBS instead of the primary antibody.

### Scanning electron microscopy

Scanning electron microscopy was processed at low accelerating voltage of the primary beam, without any coating of the sample, as described in^31^, with few modifications. The bacterial colonies were applied to silicon chips by pressing against the bacterial colony and were allowed to adhere to their surfaces for 5 min at room temperature. The samples were fixed with 5% formaldehyde in 0.1 M cacodylate buffer at 4 °C for 30 min and then washed in water and dehydrated in a series of methanol (25–50–75–100–100%) steps, each lasting 1 h at 4 °C. Samples underwent critical point drying with methanol undergoing an exchange for liquid CO_2_ in an automatized manner (CPD300 AUTO, Leica Microsystems, Austria) and were imaged with a cross-beam scanning electron microscope equipped with a Schottky field-emission cathode (Auriga 60, Carl Zeiss, Oberkochen, Germany) at 1.2 kV accelerating voltage; this process was implemented with the low-voltage field-emission scanning electron microscopy (LV-FESEM) mode and by applying the low-energy loss electron principle for generating of the highly resolved chemical contrast, as described in^32^. Images were acquired with the Everhart-Thornley electron detector (SE2 secondary electrons) and the in-lens electron detector (SE1 secondary electrons) and with the energy-selective back-scattered electron detector (EsB) directly from the sample surfaces, with no coating or contrasting applied^31,32^). The macrophages for electron microscopy were isolated from the spleen (splenocytes) of 6-week-old mice as previously described^31^. The uptake of *T. pulmonis* bacteria was performed in 10% FCS-supplemented Dulbecco’s modified Eagle’s medium without antibiotics at 37 °C for 60 min in 5% CO_2_-supplied incubator.

### Data Availability

All data generated or analysed during this study are included in this published article.
